# Poincaré and Log–Sobolev Inequalities for Mixtures

**DOI:** 10.3390/e21010089

**Published:** 2019-01-18

**Authors:** André Schlichting

**Affiliations:** Institut für Geometrie und Praktische Mathematik, RWTH Aachen, Templergraben 55, 52056 Aachen, Germany; schlichting@igpm.rwth-aachen.de

**Keywords:** Poincaré inequality, log–Sobolev inequality, relative entropy, fisher information, Dirichlet form, mixture, finite Gaussian mixtures

## Abstract

This work studies mixtures of probability measures on $R^{n}$ and gives bounds on the Poincaré and the log–Sobolev constants of two-component mixtures provided that each component satisfies the functional inequality, and both components are close in the $\chi^{2}$-distance. The estimation of those constants for a mixture can be far more subtle than it is for its parts. Even mixing Gaussian measures may produce a measure with a Hamiltonian potential possessing multiple wells leading to metastability and large constants in Sobolev type inequalities. In particular, the Poincaré constant stays bounded in the mixture parameter, whereas the log–Sobolev may blow up as the mixture ratio goes to 0 or 1. This observation generalizes the one by Chafaï and Malrieu to the multidimensional case. The behavior is shown for a class of examples to be not only a mere artifact of the method.

## 1. Introduction

A mixture of two probability measures $\mu_{0}$ and $\mu_{1}$ on $R^{n}$ is for some parameter p∈[0,1] the probability measure $\mu_{p}$ defined by

(1) $$\mu_{p}:=p\mu_{0}+\left(1-p\right)\mu_{1}.$$

Hereby, both measures $\mu_{0}$ and $\mu_{1}$ are assumed to be absolutely continuous with respect to the Lebesgue measure and their supports are nested, i.e., $\mathrm{supp}\mu_{0}\subseteq \mathrm{supp}\mu_{1}$ or $\mathrm{supp}\mu_{1}\subseteq \mathrm{supp}\mu_{0}$. Under these assumptions at least one measure is absolutely continuous to the other one

$$\mu_{0}\ll \mu_{1}\;\mathrm{or}\;\mu_{1}\ll \mu_{0}\;,$$

which implies that at least one of the measures has a density with respect to the other one

$$\;d\mu_{0}=\frac{\;d^{}\mu_{0}}{\;d\mu_{1}^{}}\;d\mu_{1}\;\mathrm{or}\;\;d\mu_{1}=\frac{\;d^{}\mu_{1}}{\;d\mu_{0}^{}}\;d\mu_{0}\;.$$

This work establishes criteria to check in a simple way under which a mixture of measures satisfies a Poincaré PI(ϱ) or log–Sobolev inequality LSI(α) with constants ϱ and α, respectively, provided that each of the components satisfies one.

**Definition** **1** (PI(ϱ) and LSI(α))**.** *A probability measure μ on $R^{n}$ satisfies the* Poincaré inequality *with constant ϱ>0, if for all functions $f:R^{n}\to R$*

PI(*ϱ*) $${\mathrm{Var}}_{\mu }[f]:=\int {\left|f-\int f\;d\mu \right|}^{2}\;d\mu \leq \frac{1}{\varrho }\int {\left|\nabla f\right|}^{2}\;d\mu \;.$$

*A probability measure μ satisfies the* log–Sobolev inequality *with constant α>0, if for all functions $f:R^{n}\to R^{+}$ holds*

LSI(*α*) $${\mathrm{Ent}}_{\mu }[f]:=\int f\;\log f\;d\mu -\int f\;d\mu \;\log(\int f\;d\mu )\leq \frac{1}{\alpha }\int \frac{{\left|\nabla f\right|}^{2}}{2f}\;d\mu \;.$$

*By the change of variable $f\mapsto f^{2}$, the log–Sobolev inequality LSI(α) is equivalent to*

(2) $${\mathrm{Ent}}_{\mu }[f^{2}]\leq \frac{2}{\alpha }\int {\left|\nabla f\right|}^{2}\;d\mu \;.$$

The question of how $\varrho_{p}$ and $\alpha_{p}$ in $\mathrm{PI}(\varrho_{p})$ and $\mathrm{LSI}(\alpha_{p})$ depend for a mixture $\mu_{p}$ on the parameter p∈[0,1] was first studied by Chafaï and Malrieu [1] for measures on $R^{n}$. The aim is to deduce simple criteria under which the measure $\mu_{p}$ (1) satisfies $\mathrm{PI}(\varrho_{p})$ and $\mathrm{LSI}(\alpha_{p})$ knowing that $\mu_{0}$ and $\mu_{1}$ satisfy $\mathrm{PI}(\varrho_{0})$, $\mathrm{PI}(\varrho_{1})$ and $\mathrm{LSI}(\alpha_{0})$, $\mathrm{LSI}(\alpha_{1})$, respectively. The approach by Chafaï and Malrieu [1] is based on a functional depending on the distribution function of the measures $\mu_{0}$ and $\mu_{1}$, which then lead to bounds on the Poincaré and log–Sobolev constant of the mixture in one dimension.

This work generalizes part of the results from Chafaï and Malrieu [1] to the multidimensional case by a simple argument. The estimates on the Poincaré and log–Sobolev constants hold for the case, when the $\chi^{2}$-distance of $\mu_{0}$ and $\mu_{1}$ is bounded (see Label (5) for its definition). For this to be true, at least one of the measures $\mu_{0}$ and $\mu_{1}$ needs to be absolutely continuous to the other, which is also a necessary condition for the mixture having connected support. The resulting bound is optimal in the scaling behavior of the mixture parameter p→0,1, i.e., a logarithmic blow-up behavior in *p* for the log–Sobolev constant, whereas the Poincaré constant stays bounded. This different behavior of the Poincaré and log–Sobolev constant was also observed in the setting of metastability in ([2], Remark 2.20).

Let us first introduce the principle for the Poincaré inequality in Section 2 and then for the log–Sobolev inequality in Section 3. Then, the procedure is illustrated on specific examples of mixtures in Section 4.

## 2. Poincaré Inequality

To keep the presentation concise, the following notation for the mean of a function $f:R^{n}\to R$ with respect to a measure μ is introduced

$$E_{\mu }\left[f\right]:=\int f\;d\mu \;.$$

In this way, the variance in PI(ϱ) and relative entropy in LSI(α) become

$${\mathrm{Var}}_{\mu }\left[f\right]=E_{\mu }[({f-E_{\mu }\left[f\right])}^{2}]=E_{\mu }\left[f\right]-{\left(E_{\mu }\left[f\right]\right)}^{2}\;\mathrm{and}\;{\mathrm{Ent}}_{\mu }\left[f\right]=E_{\mu }[f\log f]-E_{\mu }\left[f\right]\log\left(E_{\mu }\left[f\right]\right).$$

Likewise, the covariance of two functions $f,g:R^{n}\to R$ is defined by

$${\mathrm{Cov}}_{\mu }[f,g]=E_{\mu }[\left(f-E_{\mu }\left[f\right](g-E_{\mu }\left[g\right]\right)=E_{\mu }[f\;g]-E_{\mu }\left[f\right]\;E_{\mu }\left[g\right].$$

The Cauchy–Schwarz inequality for the covariance takes now the form

$${\mathrm{Cov}}_{\mu }[f,g]\leq {\mathrm{Var}}_{\mu }\left[f\right]\;{\mathrm{Var}}_{\mu }\left[g\right]\;.$$

The argument is based on an easy but powerful observation for measures $\mu_{0}$ and $\mu_{1}$ with joint support.

**Lemma** **1** (Mean-difference as covariance). *If $\mathrm{supp}\mu_{0}=\mathrm{supp}\mu_{1}$, then for any ϑ∈[0,1] and any function $f:R^{n}\to R$ holds*

(3) $$E_{\mu_{0}}\left[f\right]-E_{\mu_{1}}\left[f\right]=-\vartheta {\mathrm{Cov}}_{\mu_{0}}[f,\frac{\;d^{}\mu_{1}}{\;d\mu_{0}^{}}]+\left(1-\vartheta \right){\mathrm{Cov}}_{\mu_{1}}[f,\frac{\;d^{}\mu_{0}}{\;d\mu_{1}^{}}].$$

**Proof.**  The change of measure formula yields that the covariances above are just the difference of the expectation on the right-hand side

$${\mathrm{Cov}}_{\mu_{0}}[f,\frac{\;d^{}\mu_{1}}{\;d\mu_{0}^{}}]=E_{\mu_{0}}[f\;\frac{\;d^{}\mu_{1}}{\;d\mu_{0}^{}}]-E_{\mu_{0}}\left[f\right]\;E_{\mu_{0}}[\frac{\;d^{}\mu_{1}}{\;d\mu_{0}^{}}]=E_{\mu_{1}}\left[f\right]-E_{\mu_{0}}\left[f\right]$$

and likewise for ${\mathrm{Cov}}_{\mu_{1}}[f,\frac{\;d^{}\mu_{1}}{\;d\mu_{0}^{}}]$. □

The subsequent strategy is based on the identity (3) by using a Cauchy–Schwarz inequality to arrive at the product of two variances. Then, $\mathrm{PI}(\varrho_{0})$ or $\mathrm{PI}(\varrho_{1})$ can be applied and the parameter ϑ leaves freedom to optimize the resulting expression. This allows for proving the following theorem, which is the generalization of ([1], Theorem 4.4) to the multidimensional case for the Poincaré inequality provided $\mu_{0}$ and $\mu_{1}$ are absolutely continuous to each other.

**Theorem** **1** (PI for absolutely continuous mixtures)**.** *Let $\mu_{0}$ and $\mu_{1}$ satisfy $\mathrm{PI}(\varrho_{0})$ and $\mathrm{PI}(\varrho_{1})$, respectively, and let both measures be absolutely continuous to each other. Then, for all p∈(0,1) and q=1−p, the mixture measure $\mu_{p}=p\;\mu_{0}+q\;\mu_{1}$ satisfies $\mathrm{PI}(\varrho_{p})$ with*

(4) $$\frac{1}{\varrho_{p}}\leq \{\begin{array}{ll} \frac{1}{\varrho_{0}}\;, & \;\mathrm{if}\;\frac{\varrho_{1}}{\varrho_{0}}\geq 1+p\chi_{1},\\ \frac{1}{\varrho_{1}}\;, & \;\mathrm{if}\;\frac{\varrho_{0}}{\varrho_{1}}\geq 1+q\chi_{0},\\ \frac{p\;\chi_{1}+p\;q\;\chi_{0}\;\chi_{1}+q\;\chi_{0}}{\varrho_{0}\;p\;\chi_{1}+\varrho_{1}\;q\;\chi_{0}}\;, & \;\mathrm{else}, \end{array}$$

*where*

(5) $$\chi_{0}:={\mathrm{Var}}_{\mu_{0}}[\frac{\;d^{}\mu_{1}}{\;d\mu_{0}^{}}]\;\mathrm{and}\;\chi_{1}:={\mathrm{Var}}_{\mu_{1}}[\frac{\;d^{}\mu_{0}}{\;d\mu_{1}^{}}].$$

**Proof.**  The variance of *f* with respect to $\mu_{p}$ is decomposed to

$${\mathrm{Var}}_{\mu_{p}}[f]=p{\mathrm{Var}}_{\mu_{0}}[f]+q{\mathrm{Var}}_{\mu_{1}}[f]+p\;q{\left(E_{\mu_{0}}\left[f\right]-E_{\mu_{1}}\left[f\right]\right)}^{2}.$$

Hereby, the first two terms are just the expectation of the conditional variances. The third term is the variance of a Bernoulli random variable. Now, the mean-difference is rewritten by Lemma 1 and the square is estimated with the Young inequality introducing an additional parameter η>0

$${\left(a+b\right)}^{2}\leq \left(1+\eta \right)\;a^{2}+\left(1+\eta^{-1}\right)\;b^{2}.\;$$

Then, the Cauchy–Schwarz inequality is applied to the covariances to obtain

(6) $$\begin{array}{cc} {\mathrm{Var}}_{\mu }\left[f\right] & \leq p{\mathrm{Var}}_{\mu_{0}}\left[f\right]+q{\mathrm{Var}}_{\mu_{1}}\left[f\right]+\\ & \;+p\;q(\left(1+\eta \right)\;\vartheta^{2}\;{\mathrm{Cov}}_{\mu_{0}}^{2}[f,\frac{\;d^{}\mu_{1}}{\;d\mu_{0}^{}}]+(1+\eta^{-1})\;{\left(1-\vartheta \right)}^{2}\;{\mathrm{Cov}}_{\mu_{1}}[f,\frac{\;d^{}\mu_{0}}{\;d\mu_{1}^{}}])\\ & \leq (1+\left(1+\eta \right)\;\vartheta^{2}\;q\;\chi_{0})\;p\;{\mathrm{Var}}_{\mu_{0}}\left[f\right]+(1+(1+\eta^{-1})\;{\left(1-\vartheta \right)}^{2}\;p\;\chi_{1})\;q\;{\mathrm{Var}}_{\mu_{1}}\left[f\right]\\ & \leq \frac{1+\left(1+\eta \right)\;\vartheta^{2}\;q\;\chi_{0}}{\varrho_{0}}{\int |\nabla f|}^{2}\;p\;d\mu_{0}+\frac{1+(1+\eta^{-1})\;{\left(1-\vartheta \right)}^{2}\;p\;\chi_{1}}{\varrho_{1}}\int {\left|\nabla f\right|}^{2}\;q\;d\mu_{1}\\ & \leq \max\{\frac{1+\left(1+\eta \right)\;\vartheta^{2}\;q\;\chi_{0}}{\varrho_{0}},\frac{1+(1+\eta^{-1})\;{\left(1-\vartheta \right)}^{2}\;p\;\chi_{1}}{\varrho_{1}}\}\int |\nabla f{|}^{2}\;d\mu .\; \end{array}$$

The resulting maximum is now minimized in η>0 and ϑ∈[0,1]. To do so without loss of generality, $\varrho_{0}\geq \varrho_{1}$ is assumed. The other case can always be obtained by interchanging the roles of $\mu_{0}$ and $\mu_{1}$. If $\varrho_{0}>\varrho_{1}$, then ϑ=1 and η→0 is optimal as long as

$$\frac{1+q\;\chi_{0}}{\varrho_{0}}\leq \frac{1}{\varrho_{1}}.$$

This corresponds to the second case in (4). By symmetry, the first case follows if $\varrho_{1}\geq \varrho_{0}$.

Now, in the case $\varrho_{0}\geq \varrho_{1}$ and $\varrho_{0}\leq \left(1+q\chi_{0}\right)\varrho_{1}$, there exists by monotonicity for every ϑ∈(0,1) a unique $\eta_{*}=\eta_{*}\left(\vartheta \right)>0$ such that both terms in the max of the right-hand side in (6) are equal and hence the max is minimal. Since $q\;\chi_{0}>0$ and $p\;\chi_{1}>0$, the sum of the coefficients in the front is then given by $h\left(\vartheta \right):=\left(1+\eta \right)\vartheta^{2}+\left(1+\frac{1}{\eta }\right){\left(1-\vartheta \right)}^{2}$ in ϑ as a function of η. The minimization of *h* in ϑ∈(0,1) leads to $\vartheta_{*}=\frac{1}{1+\eta }$ and

$$h\left(\vartheta^{*}\right)=\frac{1}{1+\eta }+\frac{\eta }{1+\eta }=1$$

holds. Hence, in this case, the parameter $s=\left(1+\eta_{*}\right)\;\vartheta_{*}^{2}=\frac{1}{1+\eta_{*}}\in \left(0,1\right)$ and $\left(1+\eta_{*}^{-1}\right)\;{\left(1-\vartheta_{*}\right)}^{2}=\frac{\eta_{*}}{1+\eta_{*}}=1-s$. Thus, the problem can be rephrased: Find $s_{*}\in \left(0,1\right)$ which solves

$$\frac{1+s\;q\;\chi_{0}}{\varrho_{0}}=\frac{1+\left(1-s\right)\;p\;\chi_{1}}{\varrho_{1}}\;.$$

The solution $s_{*}$ is given by

$$s_{*}=\frac{\left(1+p\;\chi_{1}\right)\varrho_{0}-\varrho_{1}}{\varrho_{0}\;p\;\chi_{1}+\varrho_{1}\;q\;\chi_{0}}\;.$$

For this value of $s_{*}$, the value of the max in (6) is given by

$$\frac{1+s_{*}\;q\;\chi_{0}}{\varrho_{0}}=\frac{p\;\chi_{1}+\frac{\varrho_{1}}{\varrho_{0}}\;q\;\chi_{0}+\left(1+p\;,\chi_{1}\right)\;q\;\chi_{0}-\frac{\varrho_{1}}{\varrho_{0}}\;q\;\chi_{0}}{\varrho_{0}\;p\;\chi_{1}+\varrho_{1}\;q\;\chi_{0}}=\frac{p\;\chi_{1}+\;p\;q\;\chi_{0}\;\chi_{1}+q\;\chi_{0}}{\varrho_{0}\;p\;\chi_{1}+\varrho_{1}\;q\;\chi_{0}}.$$

 □

**Remark** **1.** *The constants $\chi_{0}$ and $\chi_{1}$ can be rewritten if $\mu_{0}$ and $\mu_{1}$ are mutual absolutely continuous as*

$$\chi_{0}=\int {\left(\frac{\;d\mu_{1}}{\;d\mu_{0}}\right)}^{2}\;d\mu_{0}-1=\int \frac{\;d\mu_{1}}{\;d\mu_{0}}\;d\mu_{1}-1\;\mathrm{and}\;\chi_{1}=\int {\left(\frac{\;d\mu_{0}}{\;d\mu_{1}}\right)}^{2}\;d\mu_{1}-1=\int \frac{\;d\mu_{0}}{\;d\mu_{1}}\;d\mu_{0}-1\;.$$

*This quantity is also known as*$\chi^{2}$-distance *on the space of probability measures (cf. [3]). The $\chi^{2}$-distance is a rather weak distance and therefore bounds many other probability distances. Among them is also the relative entropy. Indeed, by the concavity of the logarithm and the Jensen inequality follows*

$${\mathrm{Ent}}_{\mu_{0}}[\frac{\;d^{}\mu_{1}}{\;d\mu_{0}^{}}]=\int \log\frac{\mu_{1}}{\mu_{0}}\;d\mu_{1}\leq \log(\int \frac{\mu_{1}}{\mu_{0}}\;d\mu_{1})=\log\left(1+\chi_{0}\right)\leq \chi_{0}.$$

**Remark** **2.** *The proof of Theorem 1 shows that the expression for $\frac{1}{\varrho }$ in the last case of *(4)* can be bounded above and below by*

(7) $$\max\{\frac{1}{\varrho_{0}},\frac{1}{\varrho_{1}}\}\leq \frac{p\;\chi_{1}+p\;q\;\chi_{0}\;\chi_{1}+q\;\chi_{0}}{\varrho_{0}\;p\;\chi_{1}+\varrho_{1}\;q\;\chi_{0}}\leq \max\{\frac{1+q\;\chi_{0}}{\varrho_{0}},\frac{1+p\;\chi_{1}}{\varrho_{1}}\}\;.$$

*In the case, where $\chi_{0}=\chi_{1}=\chi$, the formula for $\varrho_{p}$*(4)* simplifies to*

(8) $$\frac{1}{\varrho_{p}}\leq \frac{1+p\;q\;\chi }{p\;\varrho_{0}+q\;\varrho_{1}}\;.$$

**Corollary** **1.** *Let $\mu_{0}\ll \mu_{1}$ and $\mu_{0}$, $\mu_{1}$ satisfy $\mathrm{PI}(\varrho_{0})$, $\mathrm{PI}(\varrho_{1})$, respectively. Then, for all p∈[0,1] with q=1−p, the mixture measure $\mu_{p}=p\;\mu_{0}+q\;\mu_{1}$ satisfies $\mathrm{PI}(\varrho_{p})$ with*

$$\frac{1}{\varrho_{p}}=\max\{\frac{1}{\varrho_{0}},\frac{1+p\;\chi_{1}}{\varrho_{1}}\}.$$

*Likewise, if $\mu_{1}\ll \mu_{0}$, then it holds*

$$\frac{1}{\varrho_{p}}=\max\{\frac{1}{\varrho_{1}},\frac{1+q\;\chi_{0}}{\varrho_{0}}\}.$$

**Proof.**  The proof is a simple consequence of Lemma 1 with ϑ=0 and a similar line of estimates as in (6). □

## 3. Log–Sobolev Inequality

In this section, a criterion for LSI(α) is established. It will be convenient to establish it in the form (2). For a function $g:R^{n}\to R^{+}$ and two probability measures $\mu_{0}$ and $\mu_{1}$, the averaged function $\bar{g}:0,1\to R^{+}$ is defined by

$$\bar{g}\left(0\right):=E_{\mu_{0}}\left[g\right]\;\mathrm{and}\;\bar{g}\left(1\right):=E_{\mu_{1}}\left[g\right].$$

Moreover, the mixture of two Dirac measures $\delta_{0}$ and $\delta_{1}$ is by slight abuse of notation denoted by $\delta_{p}:=p\;\delta_{0}+q\;\delta_{1}$ for p∈[0,1] and q=1−p. Then, the entropy of the mixture $\mu_{p}=p\;\mu_{0}+q\;\mu_{1}$ is given by

(9) $${\mathrm{Ent}}_{\mu_{p}}\left[f^{2}\right]=p{\mathrm{Ent}}_{\mu_{0}}\left[f^{2}\right]+q{\mathrm{Ent}}_{\mu_{1}}\left[f^{2}\right]+{\mathrm{Ent}}_{\delta_{p}}[\bar{f^{2}}]\;.$$

The following discrete log–Sobolev inequality for a Bernoulli random variable is used to estimate the entropy of the averaged function $\bar{f}$. The optimal log–Sobolev constant was found by Higuchi and Yoshida [4] and Diaconis and Saloff-Coste ([5], Theorem A.2.) at the same time.

**Lemma** **2** (Optimal log–Sobolev inequality for Bernoulli measures)**.** *A Bernoulli measure $\mu_{p}$ on 0,1, i.e., a mixture of two Dirac measures $\delta_{p}=p\;\delta_{0}+q\;\delta_{1}$ with p∈[0,1] and q=1−p satisfies the discrete log–Sobolev inequality*

$${\mathrm{Ent}}_{\delta_{p}}\left[g\right]\leq \frac{p\;q}{\Lambda (p,q)}{\left(g(0)-g(1)\right)}^{2}\;\mathrm{for}\;\mathrm{all}\;g:0,1\to R^{+}\;,$$

*where $\Lambda :R^{+}\times R^{+}\to R^{+}$ is the* logarithmic mean *defined by*

$$\Lambda \left(p,q\right):=\frac{p-q}{\log p-\log q},\;\mathrm{for}\;p\neq q\;\mathrm{and}\;\Lambda \left(p,p\right):=\lim_{q\to p}\Lambda \left(p,q\right)=p\;.$$

The above result allows for estimating the coarse-grained entropy in (9).

**Lemma** **3** (Estimate of the coarse-grained entropy)**.** *Let $\bar{f^{2}}:\left\{0,1\right\}\to R^{+}$ be given by $\bar{f^{2}}\left(i\right):=E_{\mu_{i}}\left[f^{2}\right]$ for i∈{0,1}. Then, for all p∈[0,1] and q=1−p,*

(10) $${\mathrm{Ent}}_{\delta_{p}}[\bar{f^{2}}]\leq \frac{p\;q}{\Lambda (p,q)}({\mathrm{Var}}_{\mu_{0}}\left[f\right]+{\mathrm{Var}}_{\mu_{1}}\left[f\right]+{\left(E_{\mu_{0}}\left[f\right]-E_{\mu_{1}}\left[f\right]\right)}^{2})\;$$

*holds.*

**Proof.**  Lemma 2 applied to ${\mathrm{Ent}}_{\delta_{p}}\left(\bar{f^{2}}\right)$ yields

(11) $$\begin{array}{cc} {\mathrm{Ent}}_{\bar{\mu }}\left(\bar{f^{2}}\right) & \leq \frac{p\;q}{\Lambda (p,q)}{\left(\sqrt{\bar{f^{2}}\left(0\right)}-\sqrt{\bar{f^{2}}\left(1\right)}\right)}^{2}\;. \end{array}$$

The square-root-mean-difference on the right-hand side of (11) can be estimated by using the fact that the function $\left(a,b\right)\mapsto {\left(\sqrt{a}-\sqrt{b}\right)}^{2}$ is jointly convex on $R^{+}\times R^{+}$. Indeed, by introducing the functions $f_{0},f_{1}:R^{n}\times R^{n}\to R^{+}$ defined by $f_{0}\left(x,y\right)=f\left(x\right)$ and $f_{1}\left(x,y\right)=f\left(y\right)$, an application of the Jensen inequality yields the estimate

(12) $$\begin{array}{cc} ({\sqrt{E_{\mu_{0}}[f^{2}]}-\sqrt{E_{\mu_{1}}[f^{2}]})}^{2} & =({\sqrt{E_{\mu_{0}\times \mu_{1}}[f_{0}^{2}]}-\sqrt{E_{\mu_{0}\times \mu_{1}}[f_{1}^{2}]})}^{2}\\ & \leq E_{\mu_{0}\times \mu_{1}}[{\left(f_{0}-f_{1}\right)}^{2}]\\ & \leq E_{\mu_{0}}[f^{2}]-2E_{\mu_{0}}[f]\;E_{\mu_{1}}[f]+E_{\mu_{1}}[f^{2}]\\ & ={\mathrm{Var}}_{\mu_{0}}\left[f\right]+{\mathrm{Var}}_{\mu_{1}}\left[f\right]+{\left(E_{\mu_{0}}\left[f\right]-E_{\mu_{1}}\left[f\right]\right)}^{2}\;. \end{array}$$

Now, a combination (11) and (12) gives (10). □

The decomposition (9) together with (10) yields that a mixture $\mu_{p}=p\;\mu_{0}+q\;\mu_{1}$ for p∈[0,1] and q=1−p satisfies

(13) $$\begin{array}{cc} {\mathrm{Ent}}_{\mu_{p}}[f^{2}] & \leq p{\mathrm{Ent}}_{\mu_{0}}[f^{2}]+q{\mathrm{Ent}}_{\mu_{1}}[f^{2}]\\ & \;+\frac{p\;q}{\Lambda (p,q)}{\mathrm{Var}}_{\mu_{0}}\left[f\right]+{\mathrm{Var}}_{\mu_{1}}\left[f\right]+{\left(E_{\mu_{0}}\left[f\right]-E_{\mu_{1}}\left[f\right]\right)}^{2}\;. \end{array}$$

The right-hand side of (13) consists of quantities, which can be estimated under the assumption that $\mu_{0}$ and $\mu_{1}$ satisfy $\mathrm{LSI}(\alpha_{0})$ and $\mathrm{LSI}(\alpha_{1})$. The following theorem provides an extension of the result ([1] Theorem 4.4) to the multidimensional case for the log–Sobolev inequality.

**Theorem** **2** (LSI for absolutely continuous mixtures)**.** *Let $\mu_{0}$ and $\mu_{1}$ satisfy $\mathrm{LSI}(\alpha_{0})$ and $\mathrm{LSI}(\alpha_{1})$, respectively, and let both measures be absolutely continuous to each other. Then, for all p∈(0,1) and q=1−p, the mixture measure $\mu_{p}=p\;\mu_{0}+q\;\mu_{1}$ satisfies $\mathrm{LSI}(\alpha_{p})$ with*

(14) $$\frac{1}{\alpha_{p}}\leq \{\begin{array}{ll} \frac{1+q\;\lambda_{p}}{\alpha_{0}}\;, & \mathrm{if}\;\frac{\alpha_{1}}{\alpha_{0}}\geq 1+p\;\lambda_{p}\left(1+\chi_{1}\left(1+q\;\lambda_{p}\right)\right)\;,\\ \frac{1+p\;\lambda_{p}}{\alpha_{1}}\;, & \mathrm{if}\;\frac{\alpha_{0}}{\alpha_{1}}\geq 1+q\;\lambda_{p}\left(1+\chi_{0}\left(1+p\;\lambda_{p}\right)\right)\;,\\ \frac{p\left(1+q\;\lambda_{p}\right)\chi_{1}+p\;q\;\lambda_{p}\;\chi_{0}\;\chi_{1}+q\left(1+p\;\lambda_{p}\right)\;\chi_{0}}{\alpha_{0}\;p\;\chi_{1}+\alpha_{1}\;q\;\chi_{0}}\;, & \;\mathrm{else}\;. \end{array}$$

*Hereby, $\chi_{0}$ and $\chi_{1}$ are given in (5) and $\lambda_{p}$ is used for the inverse logarithmic mean*

$$\lambda_{p}:=\frac{1}{\Lambda (p,q)}=\frac{\log p-\log q}{p-q}\;,\;\mathrm{for}\;p\neq \frac{1}{2}\;,\;\mathrm{and}\;\lambda_{1/2}=2\;.$$

**Proof.**  The starting point is the splitting obtained from (13). The variances and mean-difference in (13) can be estimated in the same way as in the proof (6) of Theorem 1. Additionally, the fact [6] that LSI(α) implies PI(α) is used to derive for any η>0 and any ϑ∈(0,1)

(15) $$\begin{array}{cc} {\mathrm{Ent}}_{\mu_{p}}\left[f^{2}\right] & \leq \frac{1}{\alpha_{0}}(1+q\;\lambda_{p}(1+\left(1+\eta \right)\vartheta^{2}\;\chi_{0}))\int {\left|\nabla f\right|}^{2}\;p\;d\mu_{0}\\ & \;+\frac{1}{\alpha_{1}}(1+p\;\lambda_{p}(1+\left(1+\eta^{-1}\right)\;{\left(1-\vartheta \right)}^{2}\;\chi_{1}))\int {\left|\nabla f\right|}^{2}\;q\;d\mu_{1}\\ & \leq \max\{\frac{1+q\;\lambda_{p}(1+\left(1+\eta \right)\;\vartheta^{2}\;\chi_{0})}{\alpha_{0}},\frac{1+p\;\lambda_{p}(1+\left(1+\eta^{-1}\right)\;{\left(1-\vartheta \right)}^{2}\;\chi_{1})}{\alpha_{1}}\}\int {\left|\nabla f\right|}^{2}\;d\mu_{p}. \end{array}$$

By introducing reduced log–Sobolev constants

(16) $${\tilde{\alpha }}_{0}:=\frac{\alpha_{0}}{1+q\lambda_{p}}\;\mathrm{and}\;{\tilde{\alpha }}_{1}:=\frac{\alpha_{1}}{1+p\lambda_{p}}\;,$$

as well as defining the constants ${\tilde{\chi }}_{0}$ and ${\tilde{\chi }}_{1}$ by

(17) $${\tilde{\chi }}_{0}:=\frac{\chi_{0}\lambda_{p}}{1+q\lambda_{p}}\;\mathrm{and}\;{\tilde{\chi }}_{1}=\frac{\chi_{1}\lambda_{p}}{1+p\lambda_{p}}\;,$$

the bound (15) takes the form

(18) $${\mathrm{Ent}}_{\mu_{p}}\left(f^{2}\right)\leq \max\{\frac{1+\left(1+\eta \right)\vartheta^{2}{\tilde{\chi }}_{0}}{{\tilde{\alpha }}_{0}},\frac{1+\left(1+\frac{1}{\eta }\right){\left(1-\vartheta \right)}^{2}{\tilde{\chi }}_{1}}{{\tilde{\alpha }}_{1}}\}\int {\left|\nabla f\right|}^{2}\;d\mu_{p}\;.$$

The estimate (18) has the same structure as the estimate (6), where ${\tilde{\alpha }}_{0}$, ${\tilde{\alpha }}_{i}$ play the role of $\varrho_{0}$, $\varrho_{1}$ and ${\tilde{\chi }}_{0}$, ${\tilde{\chi }}_{1}$ the roles of $\chi_{0}$, $\chi_{1}$. Hence, the optimization procedure from the proof of Theorem 1 applies to this case and the last step consists of translating the constants ${\tilde{\alpha }}_{0}$, ${\tilde{\alpha }}_{1}$ and ${\tilde{\chi }}_{0}$, ${\tilde{\chi }}_{1}$ back to the original ones. □

**Remark** **3.** *Let the bound for $\frac{1}{\alpha_{p}}$ in the last case of *(14)* be denoted by $\frac{1}{A_{p}}$. Then, the proof shows that it can be bounded above and below in the same way as in *(7)* in terms of the reduced constants *(16)* and *(17)**

$$\max\{\frac{1+q\;\lambda_{p}}{\alpha_{0}},\frac{1+p\;\lambda_{p}}{\alpha_{1}}\}\leq \frac{1}{A_{p}}\leq \max\{\frac{1+q\;\lambda_{p}\left(1+\chi_{0}\right)}{\alpha_{0}},\frac{1+p\;\lambda_{p}\left(1+\chi_{1}\right)}{\alpha_{1}}\}\;.$$

*In the case $\chi_{0}=\chi_{1}=\chi$, the simplified bound*

(19) $$\frac{1}{\alpha_{p}}\leq \frac{1+\lambda_{p}+p\;q\;\lambda_{p}\;\chi }{p\;\alpha_{0}+q\;\alpha_{1}}\;$$

*holds. The inverse logarithmic mean $\lambda_{p}=\frac{1}{\Lambda (p,q)}$ blows up logarithmically for p→0,1. Hence, even in the case χ=0, the bound *(19)* diverges logarithmically. This logarithmic divergence looks at first sight artificial, especially in comparison to *(8)* showing that the Poincaré constant is bounded. However, the next section with examples shows that this blow-up may actually occur. Hence, the bound in *(14)* is actually optimal on this level of generality.*

An analogue statement as Corollary 1 for the Poincaré constant is obtained for the lob-Sobolev constant, whose proof follows along the same lines and is omitted.

**Corollary** **2.** *Let $\mu_{0}\ll \mu_{1}$ and $\mu_{0}$, $\mu_{1}$ satisfy $\mathrm{LSI}(\alpha_{0})$ and $\mathrm{LSI}(\alpha_{1})$, respectively. Then, for any p∈(0,1) and p=1−q, the mixture measure $\mu_{p}=p\;\mu_{0}+q\;\mu_{1}$ satisfies $\mathrm{LSI}(\alpha_{p})$ with*

$$\frac{1}{\alpha_{p}}\leq \max\{\frac{1+q\;\lambda_{p}}{\alpha_{0}},\frac{1+p\;\lambda_{p}\left(1+\chi_{1}\right)}{\alpha_{1}}\}.$$

*Likewise, if $\mu_{1}\ll \mu_{0}$, then*

$$\frac{1}{\alpha_{p}}\leq \max\{\frac{1+p\;\lambda_{p}}{\alpha_{1}},\frac{1+q\;\lambda_{p}\left(1+\chi_{0}\right)}{\alpha_{0}}\}$$

*holds.*

## 4. Examples

The results of Theorems 1 and 2 are illustrated for some specific examples and also compared to the results ([1], Section 4.5), which however are restricted to one-dimensional measures. Although the criterion of Theorems 1 and 2 can only give upper bounds for the multidimensional case, when at least one of the mixture component is absolutely continuous to the other, it is still possible to obtain the optimal results in terms of scaling in the mixture parameter p→0,1.

### 4.1. Mixture of Two Gaussian Measures with Equal Covariance Matrix

Let us consider the mixtures of two Gaussians $\mu_{0}:=N\left(0,\Sigma \right)$ and $\mu_{1}:=N\left(y,\Sigma \right)$, for some $y\in R^{n}$ and a strictly positive definite covariance matrix Σ≥σId in the sense of quadratic forms for some σ>0. Then, $\mu_{0}$ and $\mu_{1}$ satisfy $\mathrm{PI}(\sigma^{-1})$ and $\mathrm{LSI}(\sigma^{-1})$ by the Bakry–Émery criterion (Theorem A1), i.e., $\varrho_{0}=\alpha_{0}=\varrho_{1}=\alpha_{1}=\sigma^{-1}$. Furthermore, the $\chi^{2}$-distance between $\mu_{0}$ and $\mu_{1}$ can be explicitly calculated as a Gaussian integral (see also [7])

$$\begin{array}{cc} \chi_{0}=\chi_{1} & =\frac{1}{{\left(2\pi \right)}^{\frac{n}{2}}\sqrt{\det\Sigma }}\int \exp(-x\cdot \Sigma^{-1}x+\frac{1}{2}\left(x-y\right)\cdot \Sigma^{-1}\left(x-y\right))\;dx-1\\ & =\exp(y\cdot \Sigma^{-1}y)\frac{1}{{\left(2\pi \right)}^{\frac{n}{2}}\sqrt{\det\Sigma }}\int \exp(-\frac{1}{2}\left(x+y\right)\Sigma^{-1}\left(x+y\right))\;dx-1\leq e^{{\left|y\right|}^{2}\;/\;\sigma }-1. \end{array}$$

Then, the bound from Theorem 1 in the form (8) yields

(20) $$\frac{1}{\varrho_{p}}\leq (1+p\;q\;(e^{{\left|y\right|}^{2}\;/\;\sigma }-1))\sigma .$$

Likewise, the log–Sobolev constant follows from Theorem 2 in the form (19) leads to

$$\frac{1}{\alpha_{p}}\leq (1+p\;q\;\lambda_{p}(e^{{\left|y\right|}^{2}\;/\;\sigma }+1))\sigma \;.$$

By noting that $pq\leq pq\lambda_{p}\leq \frac{1}{4}$, both constants stay uniformly bounded in *p*. The large exponential factor in the distance $e^{{\left|y\right|}^{2}\;/\;\sigma }$ cannot be avoided on this level of generality since the mixed measure $\mu_{p}$ has a bimodal structure leading to metastable effects ([2], Remark 2.20).

The result ([1] Corollary 4.7) deduced the following bound on $\frac{1}{\varrho_{p}}$ for the mixture of two one-dimensional standard Gaussians σ=1 in (20)

(21) $$\frac{1}{\varrho_{p}}\leq 1+{p\;q\;|y|}^{2}(\Phi \left(|y|\right)\;e^{{\left|y\right|}^{2}}+\frac{\left|y\right|}{\sqrt{2\pi }}\;e^{{\left|y\right|}^{2}\;/\;2}+\frac{1}{2})\;,$$

where $\Phi \left(a\right)=\frac{1}{\sqrt{2\pi }}\int_{-\infty }^{a}e^{-y^{2}\;/\;2}\;dy$. The elementary inequalities $e^{a^{2}}-1\leq a^{2}e^{a^{2}}$ and $\Phi \left(a\right)\geq 1+\frac{a}{\sqrt{2\pi }}e^{-a^{2}\;/\;2}$ for all a∈R show that the bound (20) is better than the bound (21) for all parameter values p∈[0,1] and |y|≥0.

Hence, this example shows that, for mixtures with components that are absolutely continuous to each other as well as whose tail behavior is controlled in terms of the $\chi^{2}$-distance, Theorems 1 and 2 even improve the bound of [1] and generalize it to the multidimensional case.

### 4.2. Mixture of a Gaussian and Sub-Gaussian Measure

Let us consider $\mu_{1}=N\left(0,\Sigma \right)$ where Σ≥σId is strictly positive definite. In addition, let the density of $\mu_{0}$ with respect to $\mu_{1}$ be bounded uniformly by some κ≥1, that is the relative density satisfies $d\mu_{0}/d\mu_{1}\leq \kappa$ almost everywhere on $R^{n}$. By the Bakry–Émery criterion (Theorem A1), $\varrho_{1}=\alpha_{1}=\frac{1}{\sigma }$ holds. Furthermore, an upper bound for $\chi_{1}$ is obtained by the assumption on the bound on the relative density

$$\chi_{1}={\mathrm{Var}}_{\mu_{1}}[\frac{\mu_{0}}{\mu_{1}}]=\int {\left(\frac{\mu_{0}}{\mu_{1}}\right)}^{2}d\mu_{1}-1\leq \kappa^{2}-1.$$

Provided that $\mu_{0}$ satisfies $\mathrm{PI}(\varrho_{0})$, the Poincaré constant of the mixture $\mu_{p}=p\;\mu_{0}+q\;\mu_{1}$ satisfies by Corollary 1 the estimate

$$\frac{1}{\varrho_{p}}\leq \max\{\frac{1}{\varrho_{0}},\left(1+p\left(\kappa^{2}-1\right)\right)\sigma \}\;.$$

Similarly, Corollary 2 provides whenever $\mu_{0}$ satisfies $\mathrm{LSI}(\alpha_{0})$ the following bound for the log–Sobolev constant of the mixture measure $\mu_{p}$

$$\frac{1}{\alpha_{p}}\leq \max\{\frac{1+q\lambda_{p}}{\alpha_{0}},\left(1+p\lambda_{p}\kappa^{2}\right)\sigma \}.$$

In this case, the logarithmic blow-up of the log–Sobolev constant cannot be ruled out for p→0,1, without any further information on $\mu_{0}$.

### 4.3. Mixture of Two Centered Gaussians with Different Variance

For $\mu_{0}=N\left(0,\mathrm{Id}\right)$ and $\mu_{1}=N\left(0,\sigma \mathrm{Id}\right)$, the Bakry–Émery criterion (Theorem A1) implies $\varrho_{0}=\alpha_{0}=1$ and $\varrho_{1}=\alpha_{1}=\sigma^{-1}$. The calculation of the $\chi^{2}$-distance can be done using the spherical symmetry and is reduced to the one dimensional integral

$$\chi_{0}=\int \frac{d\mu_{1}}{d\mu_{0}}\;d\mu_{1}-1=\frac{H^{n-1}\left(\partial B_{1}\right)}{{\left(2\pi \right)}^{\frac{n}{2}}\sigma^{n}}\int_{R^{+}}r^{n-1}e^{-(\frac{1}{\sigma }-\frac{1}{2})r^{2}}\;dr-1\;.$$

Hereby, $H^{n-1}\left(S^{n-1}\right)$ denotes the n−1-dimensional Hausdorff measure of the sphere $\partial B_{1}=x\in R^{n}:\left|x\right|=1$. The integral does only exist for σ<2. In this case, it can be evaluated and simplified. The bound for the constant $\chi_{1}$ follows by duality under the substitution $\sigma \mapsto \sigma^{-1}$ and is given by

(22) $$\chi_{0}=\left\{\begin{array}{cc} \frac{1}{{\left(\sigma (2-\sigma )\right)}^{\frac{n}{2}}}-1, & \sigma <2,\\ +\infty , & \sigma \geq 2, \end{array}\right.\;\mathrm{and}\;\chi_{1}=\left\{\begin{array}{cc} \frac{1}{{\left(\sigma^{-1}\left(2-\sigma^{-1}\right)\right)}^{\frac{n}{2}}}-1, & \sigma >\frac{1}{2},\\ +\infty , & \sigma \leq \frac{1}{2}. \end{array}\right.\;$$

If σ≤1/2, that is for $\chi_{1}=\infty$, the bound given in Corollary 1 yields

$$\frac{1}{\varrho_{p}}\leq \max\{\sigma ,1+q\chi_{0}\}=\max\{\sigma ,\left(1-q\right)+\frac{q}{{\left(\sigma (2-\sigma )\right)}^{\frac{n}{2}}}\}=p+\frac{q}{{\left(\sigma (2-\sigma )\right)}^{\frac{n}{2}}}.$$

Similarly, if σ≥2, that is, for $\chi_{0}=\infty$, the bound becomes

$$\frac{1}{\varrho_{p}}\leq \max\{1,(1+p\chi_{1})\sigma \}\leq \sigma (q+\frac{p}{{\left(\sigma^{-1}\left(2-\sigma^{-1}\right)\right)}^{\frac{n}{2}}}).$$

In the case $\frac{1}{2}<\sigma <2$, the interpolation bound (4) of Theorem 1 could be applied. However, the scaling behavior for the Poincaré constant can already be observed with the estimate (7) in Remark 2, where again, thanks to the symmetry $\sigma \mapsto \frac{1}{\sigma }$,

(23) $$\frac{1}{\varrho_{p}}\leq \{\begin{array}{ll} p+\frac{q}{{\left(\sigma (2-\sigma )\right)}^{\frac{n}{2}}}\;, & \mathrm{for}\;\sigma \leq 1\;,\\ \sigma (q+\frac{p}{{\left(\sigma^{-1}\left(2-\sigma^{-1}\right)\right)}^{\frac{n}{2}}})\;, & \mathrm{for}\;\sigma \geq 1,\; \end{array}$$

holds. Hence, the Poincaré constant stays bounded for the full range of parameter p∈[0,1] and σ>0.

In the case for the log–Sobolev constant, the bound from Corollary 2 gives

(24) $$\frac{1}{\alpha_{p}}\leq \left\{\begin{array}{cc} 1+\frac{q\lambda_{p}}{{\left(\sigma (2-\sigma )\right)}^{\frac{n}{2}}}\;, & \sigma \leq 1,\\ \sigma (1+\frac{p\lambda_{p}}{{\left(\sigma^{-1}\left(2-\sigma^{-1}\right)\right)}^{\frac{n}{2}}})\;, & \sigma \geq 1. \end{array}\right.\;$$

The bound (24) blows up logarithmically for p→0,1 in general. However, the special case σ=1, although trivially, allows for the combined bound $\frac{1}{\alpha_{p}}\leq 1+\min p,q\lambda_{p}$, which stays bounded. This behavior can be extended to the range $\sigma \in (\frac{1}{2},2)$ thanks to (22) and the interpolation bound of Theorem 2.

The result (23) can be compared with the one of ([1], Section 4.5.2), which states that, for some C>0, all σ>1 and p∈(0,1/2),

(25) $$\frac{1}{\varrho_{p,\mathrm{CM}}}\leq \sigma +Cp^{\frac{1}{\sigma -1}}\;$$

holds. In general, depending on the constant *C*, the bound (23) is better for σ small, whereas the scaling in σ is better for (25), namely linear instead of $\sigma^{\frac{3}{2}}$ as in (20).

### 4.4. Mixture of Uniform and Gaussian Measure

Let $\mu_{0}=N\left(0,\mathrm{Id}\right)$ and $\mu_{1}=\frac{1}{H^{n}\left(B_{1}\right)}1_{B_{1}}$ with $B_{1}$ the unit ball around zero. Then, $\varrho_{0}=1$ holds by the Bakry–Émergy criterion (Theorem A1) and $\varrho_{1}\geq \frac{\pi^{2}}{\mathrm{diam}{\left(B_{1}\right)}^{2}}=\frac{\pi^{2}}{4}$ by the result of [8]. Furthermore, since $\mu_{1}\ll \mu_{0}$, the $\chi^{2}$-distance between $\mu_{0}$ and $\mu_{1}$ becomes thanks to the spherical symmetry

(26) $$\chi_{0}+1=\int {\left(\frac{\mu_{1}}{\mu_{0}}\right)}^{2}\;d\mu_{0}=\frac{{\left(2\pi \right)}^{\frac{n}{2}}}{H^{n}{\left(B_{1}\right)}^{2}}\int_{B_{1}}e^{\left|x\right|/2}\;dx=\frac{{\left(2\pi \right)}^{\frac{n}{2}}H^{n-1}\left(\partial B_{1}\right)}{H^{n}{\left(B_{1}\right)}^{2}}\int_{0}^{1}r^{n-1}e^{r^{2}/2}dr.$$

The volume $H^{n}\left(B_{1}\right)$ and the surface area $H^{n-1}\left(\partial B_{1}\right)$ of the *n*-sphere satisfy the following relations

(27) $$\frac{H^{n-1}\left(\partial B_{1}\right)}{H^{n}\left(B_{1}\right)}=n\;\mathrm{and}\;\frac{{\left(2\pi \right)}^{\frac{n}{2}}}{H^{n}\left(B_{1}\right)}=2^{\frac{n}{2}}\Gamma (\frac{n}{2}+1)=:g_{n}.$$

The integral on the right-hand side in (26) can be bounded below by $\frac{1}{n}$ and above by $\frac{\sqrt{e}}{n}$, which altogether yields

$$g_{n}\leq \chi_{0}+1\leq \sqrt{e}g_{n}.$$

Corollary 1 implies that the Poincaré constant of the mixture $\mu_{p}=p\;\mu_{0}+q\;\mu_{1}$ satisfies

(28) $$\frac{1}{\varrho_{p}}\leq \max\{\frac{1}{\varrho_{1}},1+q\chi_{0}\}\leq p+q\sqrt{e}\;g_{n},$$

where the last inequality follows from $\frac{4}{\pi^{2}}\leq p+q\;\sqrt{e}\;g_{n}$ for n≥1 and all p∈[0,1].

The estimate of the log–Sobolev constant uses $\alpha_{0}=1$ by the Bakry–Émergy criterion (Theorem A1) and $\alpha_{1}\geq \frac{2}{e}$ from (A1). Then, Corollary 2 yields the bound

(29) $$\begin{array}{c} \frac{1}{\alpha_{p}}\leq \max\{\frac{1+p\lambda_{p}}{\alpha_{1}},\frac{1+q\lambda_{p}\left(1+\chi_{0}\right)}{\alpha_{0}}\}\leq \max\{\frac{(1+p\lambda_{p})e}{2},1+q\lambda_{p}\sqrt{e}g_{n}\}. \end{array}$$

There is a logarithmically blow-up of the bound for p→0,1.

The blow-up for p→1 is artificial, which can be shown by a combination Bakry–Émery criterion and the Holley–Stroock perturbation principle. To do so, the Hamiltonian of $\mu_{p}$ is decomposed into a convex function and some error term

(30) $$\begin{array}{cc} H_{p}\left(x\right) & :=-\log\mu_{p}\left(x\right)=-\log(\frac{p}{{\left(2\pi \right)}^{\frac{n}{2}}}e^{-\frac{{\left|x\right|}^{2}}{2}}+\frac{1-p}{H^{n}\left(B_{1}\right)}{𝟙}_{B_{1}\left(0\right)}\left(x\right))\\ & =-\log(e^{-\frac{{\left|x\right|}^{2}}{2}+\frac{1}{2}}+\frac{1-p}{p}\frac{{\left(2\pi \right)}^{\frac{n}{2}}}{H^{n}\left(B_{1}\right)}\sqrt{e}{𝟙}_{B_{1}\left(0\right)}\left(x\right))+C_{p,n}\\ & =\frac{{\left|x\right|}^{2}-1}{2}-\psi_{p}\left(x\right)+{\tilde{C}}_{p,n}, \end{array}$$

where

$$\psi_{p}\left(x\right):=(\log(e^{-\frac{{\left|x\right|}^{2}}{2}+\frac{1}{2}}+\frac{1-p}{p}\frac{{\left(2\pi \right)}^{\frac{n}{2}}}{H^{n}\left(B_{1}\right)}\sqrt{e})+\frac{{\left|x\right|}^{2}-1}{2}){𝟙}_{B_{1}\left(0\right)}\left(x\right).$$

The function $\psi_{p}$ is radially monotone towards the boundary of $B_{1}$, which yields for |x|→1 the bound

(31) $$0\leq \psi_{p}\left(x\right)\leq \log(1+\frac{1-p}{p}\frac{{\left(2\pi \right)}^{\frac{n}{2}}}{H^{n}\left(B_{1}\right)}\sqrt{e})\;.$$

From (30), the Hamiltonian $H_{p}$ is compared with the convex potential $\frac{{\left|x\right|}^{2}-1}{2}$ with the bound (31) on the perturbation $\psi_{p}$. This together yields, by the Bakry–Émergy criterion (Theorem A1) and the Holley–Stroock perturbation principle (Theorem A2), the $\mu_{p}$ satisfies $\mathrm{PI}({\tilde{\varrho }}_{p})$ and $\mathrm{LSI}({\tilde{\alpha }}_{p})$ with

(32) $$\frac{1}{{\tilde{\varrho }}_{p}}\leq \frac{1}{{\tilde{\alpha }}_{p}}\leq 1+\frac{1-p}{p}\sqrt{e}\;g_{n},$$

where $g_{n}$ is the same constant as in (27). This bound only blows up for p→0. However, the blow-up is like $\frac{1}{p}$. Furthermore, the bound on the Poincaré constant is worse than the one from (28). Therefore, both approaches need to be combined.

The combination of the bounds obtained in (29) and (32) results in the improved bound

(33) $$\frac{1}{\alpha }\leq C_{n}\left(1+q\lambda_{p}g_{n}\right),\;\;\mathrm{with}\;C_{n}\;\mathrm{some}\;\mathrm{universal}\;\mathrm{constant},$$

which only logarithmically blows up for p→0.

This example shows that the Poincaré constant and log–Sobolev constant may have different scaling behavior for p→0. Indeed, Ref. [1] shows, for this specific mixture in the one-dimensional case that the log–Sobolev constant can be bounded below by

$$C|\log p|\leq \frac{1}{\alpha },$$

for *p* small enough and a constant *C* independent of *p*. In one dimension, lower bounds are accessible via the functional introduced by Bobkov–Götze [9]. Hence, the bound (33) is optimal in the one-dimensional case, which strongly indicates also optimality for the higher dimension case in terms of scaling in the mixture ration *p*.

To conclude, the Bakry–Émery criterion in combination with the Holley–Stroock perturbation principle is effective for detecting blow-ups of the log–Sobolev constant for mixtures, but has, in general, the wrong scaling behavior in the mixing parameter *p*. On the other hand, the criterion presented in Theorem 2 provides the right scaling of the blow-up but may give artificial blow-ups, if the components of the mixture become singular in the sense of the $\chi^{2}$-distance.

## 5. Conclusions

Recently, the investigation of mixtures can be found in many different applications, and the main results of this work may be useful to the investigation of asymmetric Kalman filter estimates [10], the study of asymmetric mixtures in Marine Biology [11], Econometrics [12], Gradient-quadratic and fixed-point iteration algorithms [7] and estimates of multivariate Gaussian mixtures [13].

Theorems 1 and 2 provide a simple estimate of the Poincaré and log–Sobolev constants of a two-component mixture measure $\mu_{p}=p\;\mu_{0}+q\;\mu_{1}$ if the $\chi^{2}$-distance of $\mu_{0}$ and $\mu_{1}$ is bounded and each of the components satisfies a Poincaré or log–Sobolev inequality. Section 4 reviews several examples with the following findings:

- For mixtures with components that are mutually absolutely continuous and whose tail behavior is mutually controlled in terms of the $\chi^{2}$-distance, Theorems 1 and 2 are very effective.
- If only one of the components is absolutely continuous to the other one with bounded density, then it is still possible to obtain a bound on the Poincaré and log–Sobolev constant. However, the log–Sobolev constant blows up logarithmically in the mixture parameter *p* approaching 0 or 1. It is shown for specific examples that this blow-up is at least for one limit p→0 or p→1 not artificial due to the applied method.
- A necessary condition for the finiteness of the $\chi^{2}$-distance between two measures is that at least one of the measures $\mu_{0}$ and $\mu_{1}$ is absolutely continuous to the other one, which in particular provides a mixture with connected support. This condition is too strong since one can easily decompose a measure into a mixture, where the joint support of the components is a null set. In this case, the present approach would not be helpful, even though the mixture may still satisfy both functional inequalities.

Future work could overcome the limits of the present approach by revisiting the crucial ingredient for both the Poincaré and log–Sobolev inequality, which was the representation of the mean-difference in Lemma 1 regarding covariances. Formula (3) from Lemma 1 applies only in the case where both measures are mutually absolutely continuous. However, the idea of an interpolation bound can be generalized to suitable weighted Sobolev spaces. For this, since $\mu_{0},\mu_{1}\ll \mu_{p}$ for all p∈(0,1), one can formally write and estimate

(34) $$E_{\mu_{0}}\left[f\right]-E_{\mu_{1}}\left[f\right]={\mathrm{Cov}}_{\mu_{p}}[f,\frac{\;d^{}\mu_{0}}{\;d\mu_{p}^{}}-\frac{\;d^{}\mu_{1}}{\;d\mu_{p}^{}}]\leq {\left\|f\right\|}_{{\dot{H}}^{1}\left(\mu_{p}\right)}{\left\|\frac{\;d^{}\mu_{0}}{\;d\mu_{p}^{}}-\frac{\;d^{}\mu_{1}}{\;d\mu_{p}^{}}\right\|}_{{\dot{H}}^{-1}\left(\mu_{p}\right)}\;.$$

Hereby, ${\dot{H}}^{1}\left(\mu_{p}\right)$ is the homogeneous weighted ${\dot{H}}^{1}$ space with norm $f_{H^{1}\left(\mu_{p}\right)}^{2}:=\int {\left|\nabla f\right|}^{2}\;d\mu_{p}$ and ${\dot{H}}^{-1}\left(\mu_{p}\right)$ is its dual space with norm

$${\left\|\omega \right\|}_{{\dot{H}}^{-1}\left(\mu_{p}\right)}^{2}:=\underset{f\in {\dot{H}}^{1}\left(\mu_{p}\right)}{\sup}\{2{\langle f,\omega \rangle }_{\mu_{p}}-{\left\|f\right\|}_{H^{1}\left(\mu_{p}\right)}^{2}\}\;.$$

The representation (34) is fruitful to many more applications in which the components of the mixture do not need to be absolutely continuous. Similar ideas for estimating mean-differences were successfully applied in the metastable setting [2,14], in which suitable bounds on the ${\dot{H}}^{-1}$-norm are obtained. In this regard, the bound (34) promises many interesting new insights for future studies. 

## Appendix A. Bakry–Émery Criterion and Holley–Stroock Perturbation Principle

Two classical conditions for Poincaré and log–Sobolev inequalities are stated in this part of the appendix. The *Bakry–Émery criterion* relates the convexity of the Hamiltonian of a measure and positive curvature of the underlying space to constants for the Poincaré and log–Sobolev inequalities. Although the result is classical for the case of $R^{n}$, the result for general convex domain was established in ([16], Theorem 2.1).

**Theorem** **A1** (Bakry–Émery criterion ([17] Proposition 3, Corollary 2), ([16], Theorem 2.1))**.** *Let $\Omega \subset R^{n}$ be convex and let H:Ω→R be a Hamiltonian with Gibbs measure $\mu \left(\;dx\right)=Z_{\mu }^{-1}e^{-H(x)}{𝟙}_{\Omega }\left(x\right)\;dx$ and assume that $\nabla^{2}H\left(x\right)\geq \kappa >0$ for all x∈suppμ. Then, μ satisfies PI(ϱ) and LSI(α) with*

ϱ≥κandα≥κ.

The second condition is the *Holley–Stroock perturbation principle*, which allows to show Poincaré and log–Sobolev inequalities for a very large class of measures.

**Theorem** **A2** (Holley–Stroock perturbation principle ([18], p. 1184))**.** *Let $\Omega \subset R^{n}$ and H:Ω→R and $\psi :\Omega \to R^{n}$ be a bounded function. Let μ and $\tilde{\mu }$ be the Gibbs measures with Hamiltonian H and H+ψ, respectively*

$$\mu \left(\;dx\right)=\frac{1}{Z_{\mu }}e^{-H(x)}{𝟙}_{\Omega }\left(x\right)\;dx\;\mathrm{and}\;\tilde{\mu }\left(\;dx\right)=\frac{1}{Z_{\tilde{\mu }}}e^{-H(x)-\psi (x)}{𝟙}_{\Omega }\left(x\right)\;dx\;.$$

*Then, if μ satisfies PI(ϱ) and LSI(α), then $\tilde{\mu }$ satisfies $\mathrm{PI}(\tilde{\varrho })$ and $\mathrm{LSI}(\tilde{\alpha })$, respectively. Hereby, the constants satisfy*

$$\tilde{\varrho }\geq e^{-{\mathrm{osc}}_{\Omega }\psi }\varrho \;\mathrm{and}\;\tilde{\alpha }\geq e^{-{\mathrm{osc}}_{\Omega }\psi }\alpha \;,$$

*where ${\mathrm{osc}}_{\Omega }\psi :=\sup_{\Omega }\psi -\inf_{\Omega }\psi$.*

Proofs relying on semigroup theory of Theorems A1 and A2 can be found in the exposition by Ledoux ([6], Corollary 1.4, Corollary 1.6 and Lemma 1.2).

**Example** **A1** (Uniform measure on the ball)**.**
*The measure $\mu_{1}=\frac{1}{H^{n}\left(B_{1}\right)}{𝟙}_{B_{1}}$, with $B_{1}$ is the unit ball around zero, satisfies $\mathrm{LSI}(\alpha_{1})$ with*

(A1) $$\alpha_{1}\geq \frac{2}{e}\;.$$

*The proof compares the measure $\mu_{1}$ with a family of measures*

$$\nu_{\sigma }\left(\;dx\right)=\frac{1}{Z_{\sigma }}\exp(-{\sigma |x|}^{2}+\frac{\sigma }{2})𝟙\left(x\right)\;dx\;\mathrm{for}\;\sigma >0\;.$$

*Then, it holds that $\nu_{\sigma }$ satisfies LSI(2σ) by the Bakry–Émery criterion (Theorem A1). Moreover, it holds that ${\mathrm{osc}}_{x\in B_{1}}\left|-{\sigma |x|}^{2}+\sigma /2\right|=\frac{\sigma }{2}$ and hence $\mu_{1}$ satisfies $\mathrm{LSI}(2\sigma e^{-\sigma })$ by the Holley–Stroock perturbation principle (Theorem A2) for all σ>0. Optimizing the expression $2\sigma e^{-\sigma }$ in σ gives the bound *(A1)*.*
